# Supplementary material for: Combating acquired resistance to MAPK inhibitors in melanoma by targeting Abl1/2-mediated reactivation of MEK/ERK/MYC signaling
Source: Nat Commun. 2020 Oct 29;11:5463. doi: 10.1038/s41467-020-19075-3 (PMC7596241; doi:10.1038/s41467-020-19075-3)
Supplement: Supplementary file 1 — Supplementary Information [file 41467_2020_19075_MOESM1_ESM.pdf]

Supplementary Figures and Tables

COMBATING ACQUIRED RESISTANCE TO MAPK INHIBITORS IN MELANOMA BY TARGETING ABL1/2-MEDIATED REACTIVATION OF MEK/ERK/MYC SIGNALING

Tripathi et al.

Supplementary Figure 1

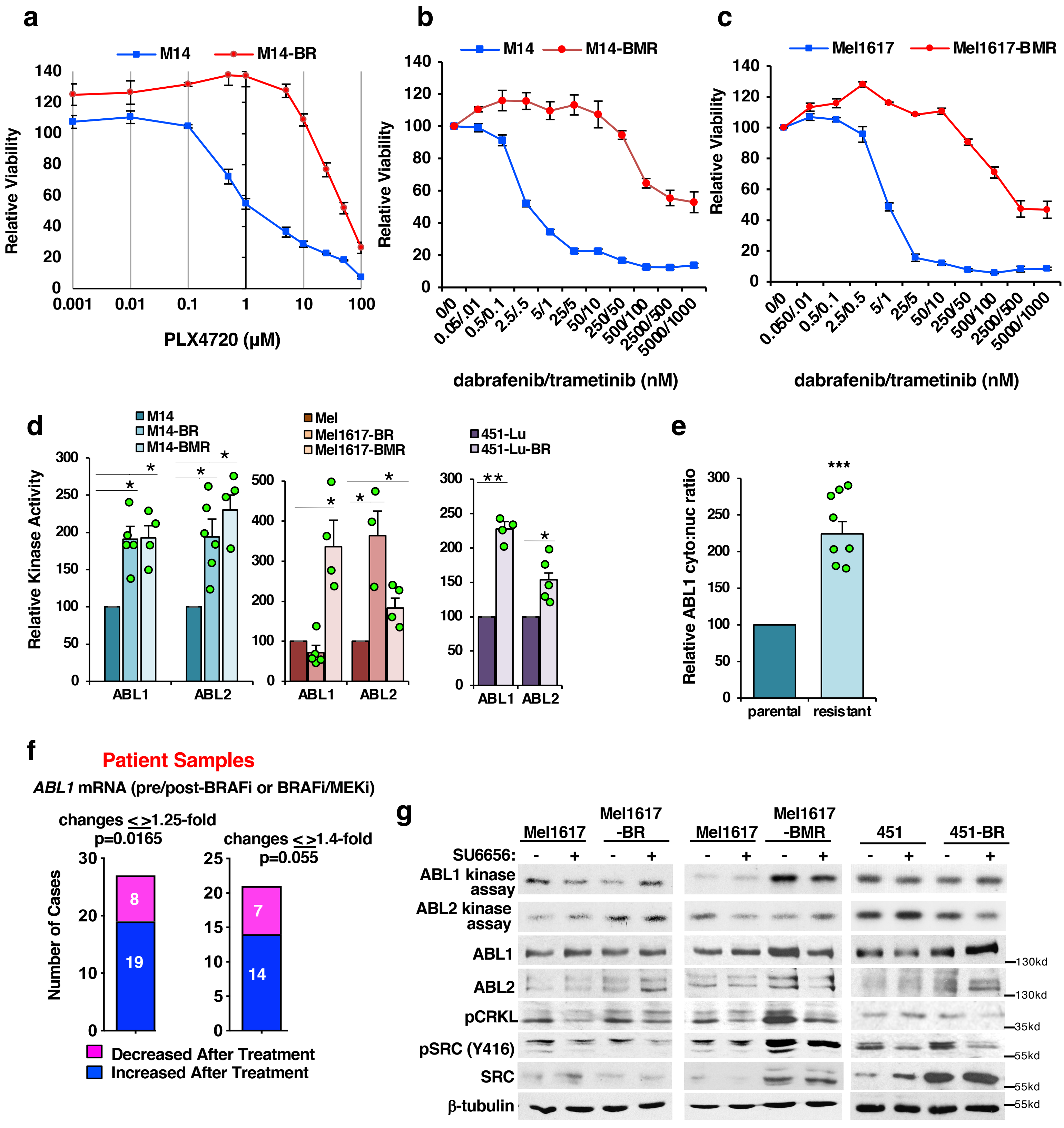

Supplementary Figure 1. ABL kinases are activated during resistance. (a-c) Parental and resistant lines were treated with various concentrations of BRAFi (PLX4720; M14-BR; a), or BRAFi/MEKi (dabrafenib/trametinib-D/T; b,c), and cell viability assessed by MTT assay after 72h. Graphs are Mean±SEM for n=3 biological replicates. Some errorbars are too small to be visualized. **(d)** Quantitation of kinase assays shown in Fig. 1a (and biological replicates). Mean±SEM. M14-BR: ABL1-n=5, ABL2-n=6; M14-BMR: ABL1, ABL2-n=4; Mel1617-BR: ABL1-n=5, ABL2-n=4; Mel1617-BMR: ABL1, n=4 ABL2, n=3; 451-Lu-BR: ABL1-n=4; ABL2-n=5. ***p<0.001, **p≤0.01, *p<0.05 using one sample t-tests (2-sided) followed by Holm's method for multiple comparisons. Actual p-values (left->right): graph #1: 0.012, 0.015, 0.016, 0.015; graph #2: 0.043, 0.031, 0.043; graph#3: 0.003, 0.024. **(e)** Cytoplasmic/nuclear ratios were obtained for all resistant lines and the results compared to parental lines. Mean±SEM. ***p=0.000156 using a one sample t-test (2-sided). **(f)** Graph of fold changes using the indicated cut-off values following analysis of *ABL1* mRNA expression in RNAseq and microarray datasets (see Fig. 1d). Numbers in graph represent number of cases. The indicated p-values are from 2-sided binomials. **(g)** Cells were treated as in Fig. 1e, and lysates subjected to kinase assay (top 2 panels) and western blots (bottom panels).

Supplementary Figure 2

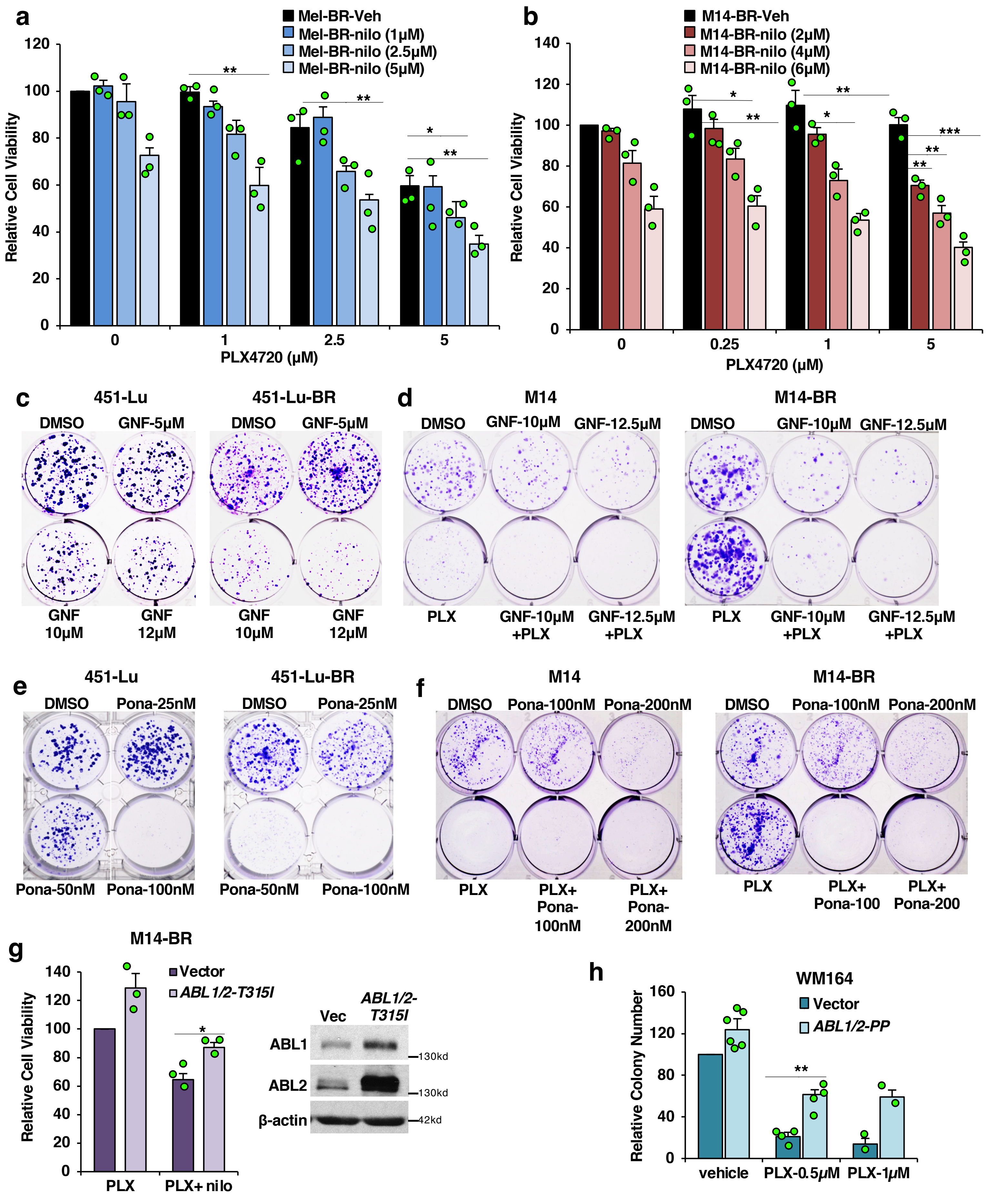

Supplementary Figure 2. ABL1/2 drive BRAFi resistance. (a,b) CellTiter Glo (CTG) viability assays for BRAFi-resistant lines treated with 3 doses of nilotinib and BRAFi (PLX4720). Results are Mean±SEM for 3 independent experiments performed in triplicate. *p<0.05, **p≤0.01, p<0.001 using two sample t-tests (2-sided). Actual p-values (left->right): (a) 0.0072, 0.0027, 0.039, 0.0068. (b) 0.045, 0.0048, 0.016, 0.0029, 0.0023, 0.001, 0.00016. **(c-f)** Colony assays utilizing the highly specific ABL1/2 inhibitor, GNF-5, a 3rd generation ABL1/2 inhibitor, ponatinib (pona), and/or BRAFi, PLX4720 (5μM). Cells were treated with drugs for 13 days (GNF) or 7 days (ponatinib), and colonies stained with crystal violet on d13 (GNF) or d10 (ponatinib). **(g)** M14-BR cells expressing vector or nilotinib-resistant forms of *ABL1* and *ABL2* (T315I) were treated with PLX4720 (2.5μM) in the absence or presence of nilotinib (2.5μM), and cell viability assessed by Cell Titer Glo after 72h. Blots on the right indicate expression levels prior to plating for CTG. Mean±SEM shown for n=3 independent experiments. The ability of ABL1/2-T315I to prevent nilotinib from reducing viability was assessed with a two-sample t-test (2-sided); *p=0.013. **(h)** Colonies for experiment shown in Fig. 2h were manually counted in Photoshop, and Mean±SEM shown for n=4 (0.5μM) or n=2 (1μM) independent experiments. **p=0.0017 (0.5μM) using a two-sample t-test (2-sided).

Supplementary Figure 3

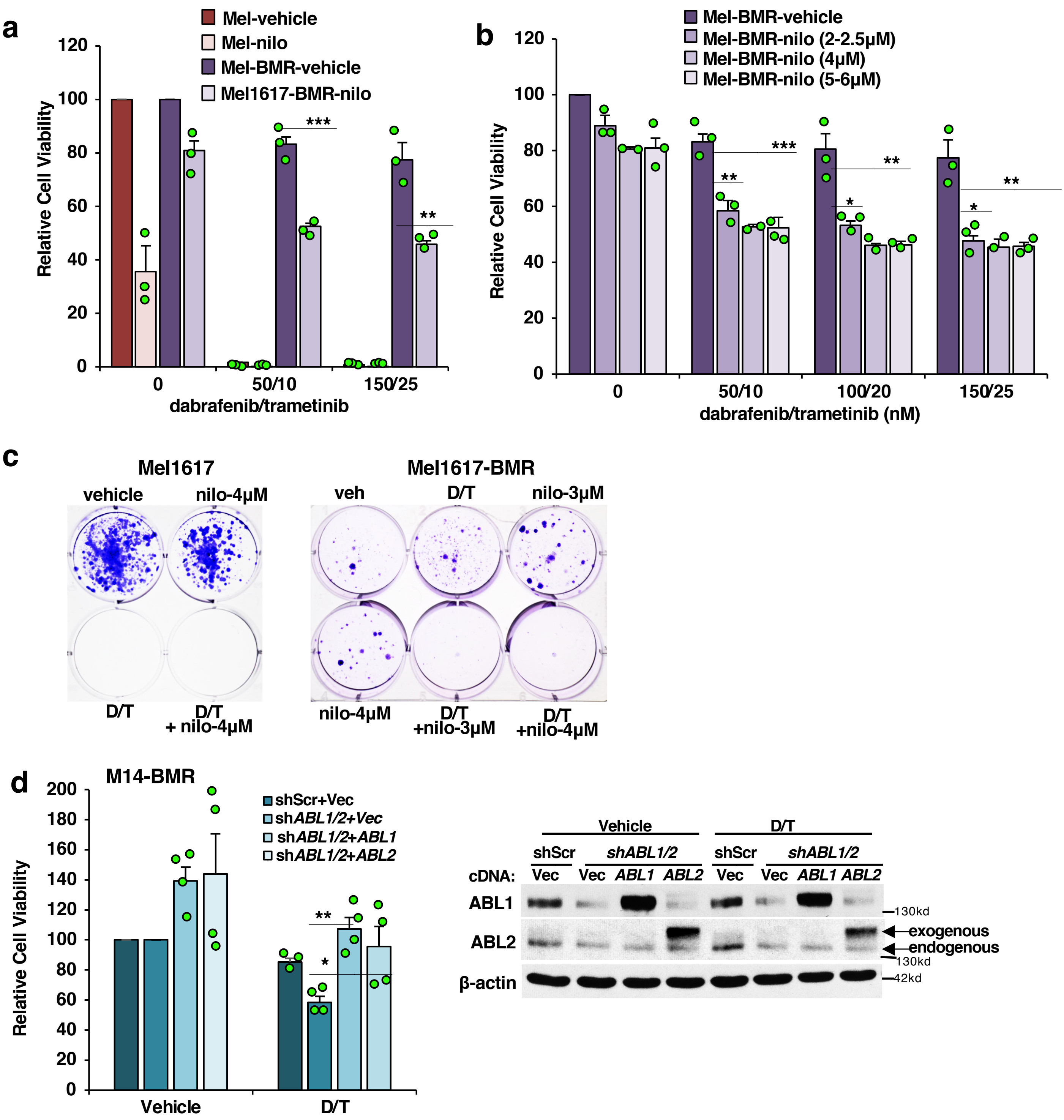

Supplementary Figure 3. Blocking ABL1/2 activation reverses resistance to BRAF+MEK inhibitors. (a,b) CellTiter Glo viability assays for parental Mel1617 and BRAFi/MEK-resistant Mel1617-BMR cells treated for 72h. **(a)** Parental and BMR cells treated with nilotinib (5 or 6μM) and the indicated doses of BRAFi (dabrafenib; D) + MEKi (trametinib; T). **(b)** Three doses of nilotinib and D/T for Mel1617-BMR cells are shown. Results are Mean±SEM for 3 independent experiments performed in triplicate (except for nilotinib 4μM-100/20 and 4μM-150/25 doses, n=2). *$p<0.05$, **$p\leq0.01$, $p<0.001$ using one sample t-tests (2-sided). Actual p-values (left->right): (a) 0.00048, 0.0088. (b) 0.0014, 0.00048, 0.011, 0.0036, 0.012, 0.0088. **(c)** Colony formation assays. Cells were plated, treated with vehicle, nilotinib (nilo), D/T (100nM/20nM) or the triple combination for 14 days, and stained as described in Fig. 3c. **(d)** M14-BMR cells expressing scrambled shRNA (shScr) or IPTG-inducible shRNA targeting *ABL1/2* (sh*ABL1/2*), were pre-treated with IPTG for 4d (1mM). Cells were then replated into 6-wells, and scrambled shRNA-expressing cells transfected with vector (pSRα), whereas *ABL1/2*-shRNA-expression cells were transfected with either vector (pSRα), wild-type *ABL1,* or wild-type *ABL2* (all in the presence of IPTG). The next day, transfected cells were harvested and replated into 96-wells (for viability assays) or 60mm dishes (for western blots) in the presence of IPTG. Ninety-six wells (viability) were treated the following day with DMSO (vehicle) or D/T (50nM/10nM) in the presence of IPTG, and viability assessed by CellTiter Glo after 72h. Graphs are Mean±SEM for n=4 biological replicates. **$p=0.0013$, *$p=0.039$ using two-sample, 2-sided t-tests. Cells in 60mm dishes were treated with DMSO (vehicle) or D/T (50nM/10nM) for 24h (since 72h induces cell death in cells expressing *ABL1/2 shRNA*), two days after plating, and lysates blotted with the indicated antibodies (a representative experiment of n=4 is shown). Western blot control lysates were harvested on the same day as the corresponding viability assay.

Supplementary Figure 4

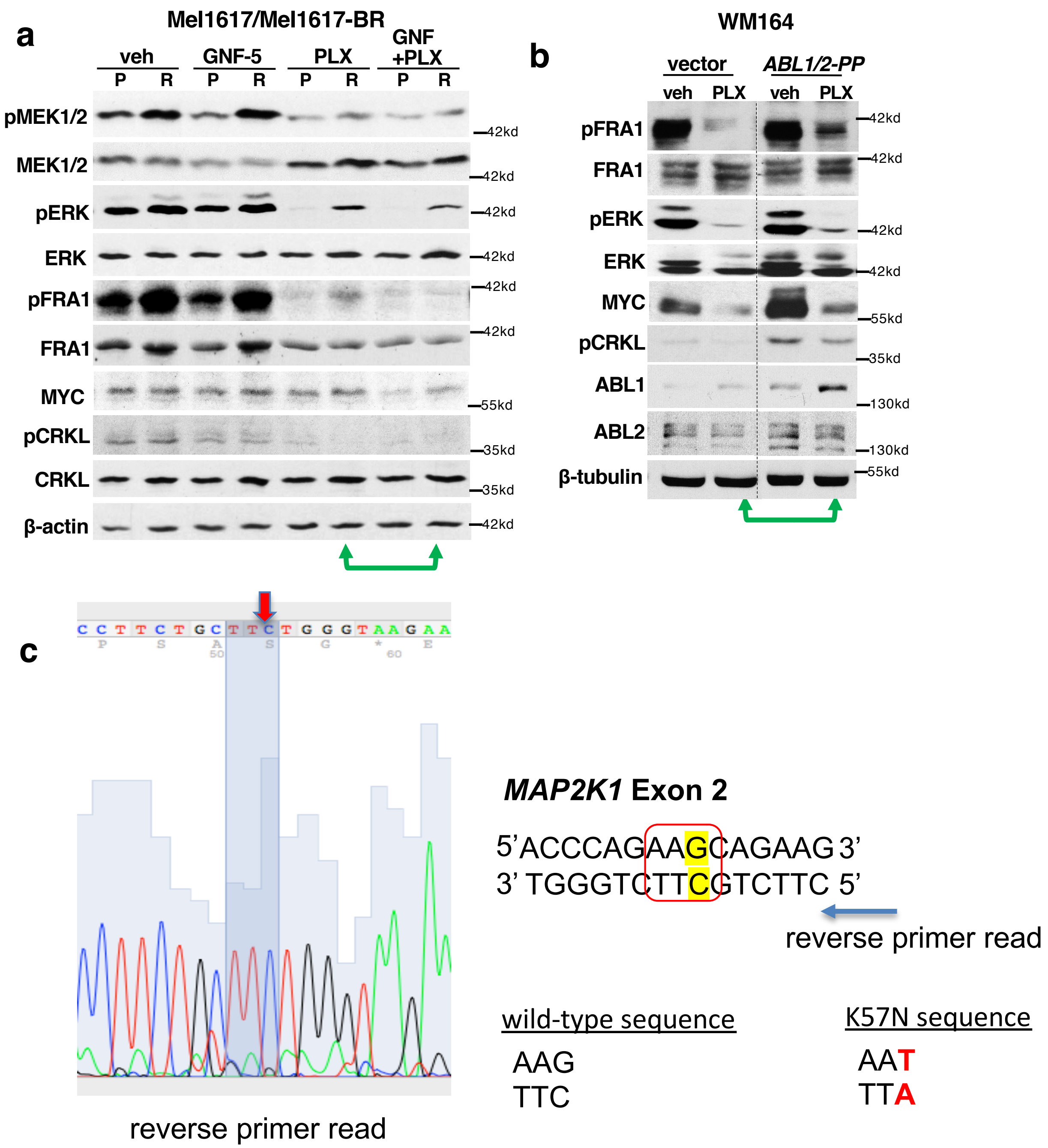

Supplementary Figure 4. ABL1/2 drive BRAFi resistance. (a) Parental and BRAFi-resistant cells were treated with vehicle, GNF-5 (10μM), -/+ BRAFi, PLX4720 (2.5μM) for 24h, and lysates blotted. A representative experiment from n=3 independent experiments is shown. **(b)** BRAF-sensitive WM164 melanoma cells, which lack endogenous activated ABL1/2 and harbor BRAF-V600E, were engineered to express either vector or constitutively active forms of *ABL1* and *ABL2* (PP). Phospho-CRKL (ABL1/2 substrate) blots demonstrate that ABL1/2-PP are highly activated despite low level expression. Cells were treated with PLX4720 (0.5μM-24h for all blots except FRA1/ERK-0.1μM-6h), and lysates blotted with the indicated antibodies. A representative experiment from n=3 independent experiments is shown. **(c)** Sanger sequencing of *MAP2K1* exon 2 from 451-Lu-BR cells viewed on 4 Peaks software. A screenshot of codon K57 is shown (wild-type in 451-Lu-BR).

Supplementary Figure 5

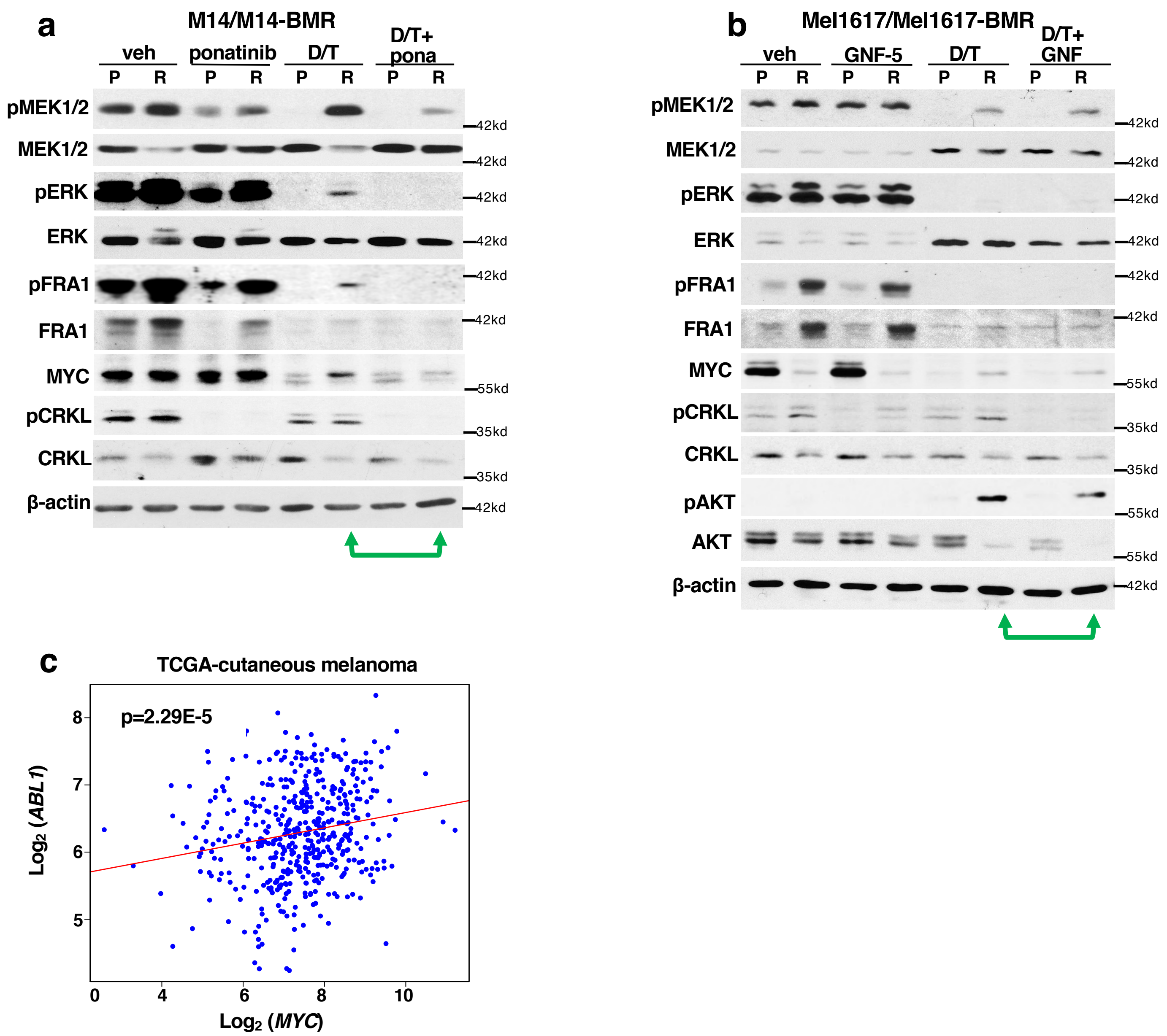

Supplementary Figure 5. ABL1/2 drive resistance to BRAF/MEK inhibitors. Parental and BRAFi/MEKi-resistant M14-BMR (a) or Mel1617-BMR (b) cells were treated with vehicle, another independent ABL1/2 inhibitor, ponatinib (100nM; a) or the less potent but highly specific ABL1/2 inhibitor, GNF-5 (10μM, b) in the absence or presence of BRAFi/MEKi (dabrafenib/trametinib-D/T (100nM/20nM-a; 150nM/25nM- b) for 24h, and lysates subjected to western blot. Representative experiments from n=4 (a) or n=3 (b) independent experiments are shown. **(c)** TCGA Skin Cutaneous Melanoma data were normalized, and Spearman's coefficient used to assess gene correlations. Correlation coefficient=0.194, 95% confidence limits (0.108, 0.283), p=2.29E-5. n=468

Supplementary Figure 6

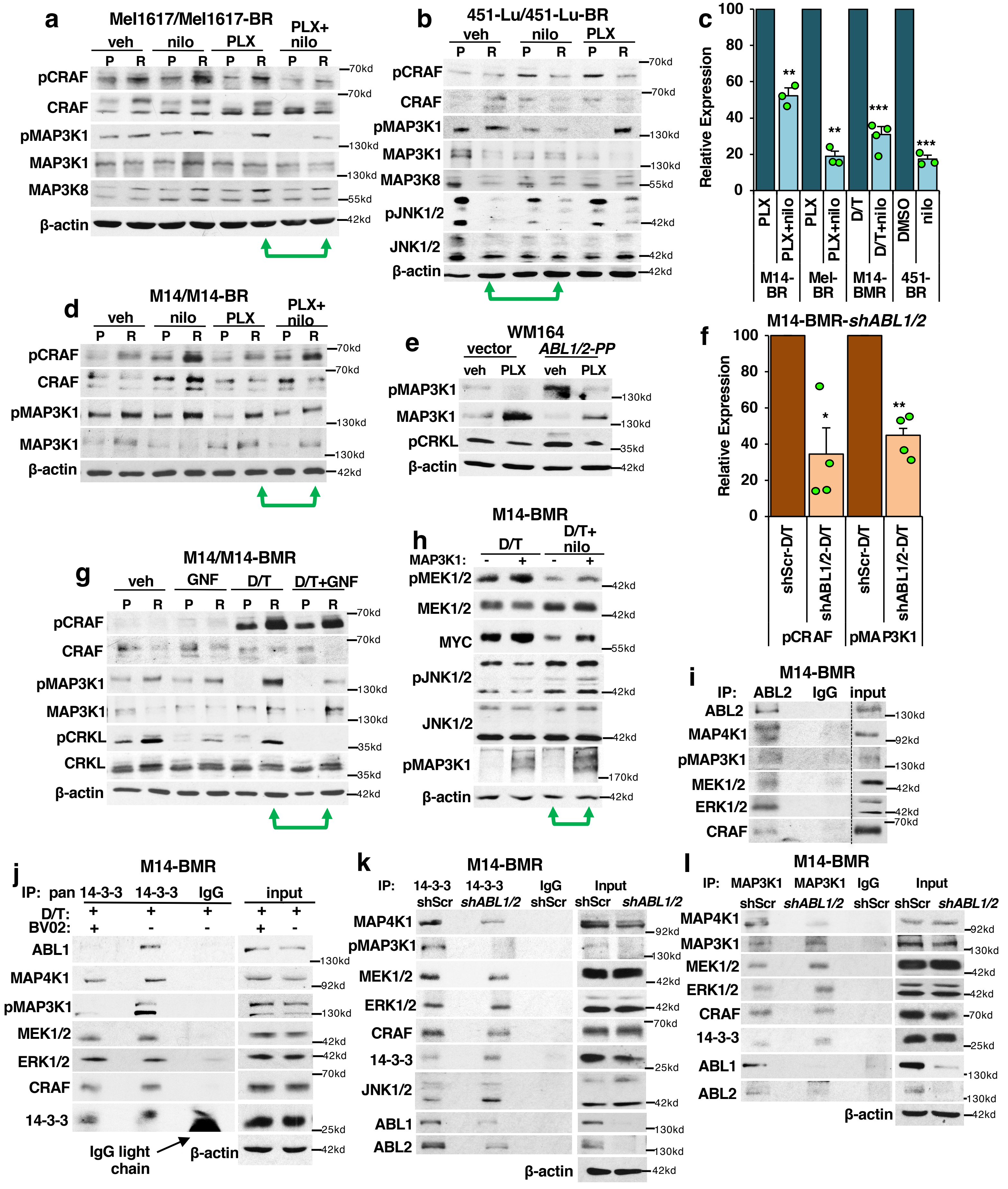

Supplementary Figure 6. ABL1/2 activate MAP3K1. (a,b,d,g) Cells were treated with the indicated drugs as in Figs. 4,5 for 24h, and resulting lysates blotted with antibodies. Drug doses are as follows: nilotinib (2.5μM), GNF-5 (10μM), PLX (1μM-M14, 451-Lu; 2.5μM-Mel1617), D/T (150nM/25nM). **(c)** Quantitation of blots shown in Fig. 6c and Supplementary Fig. 6a,b,d together with replicate experiments. Mean±SEM. Biological replicates n-values are as follows. M14-BR, Mel1617-BR, n=3; M14-BMR, n=4, 451-Lu-BR, n=3. Actual p-values (left->right) from 2-sided, one sample t-tests are: 0.0088, 0.0012, 0.00055, 0.00067 **(e)** WM164 cells expressing vector or constitutively active *ABL1*/*2*-PP were treated with PLX4720 (0.5μM-24h), and lysates blotted with the indicated antibodies. **(f)** Quantitation of blots in Fig. 6d. Mean±SEM for n=4. *$p<0.05$, **$p\leq0.01$ using one sample t-tests (2-sided). Actual p-values (left->right): 0.02, 0.0026. **(h)** M14-BMR cells were transiently transfected with vector or MAP3K1, cells replated, drug-treated (D/T=50nM/10nM; nilo=2.5μM) for 16-24h, and lysates blotted with the indicated antibodies. **(i)** ABL2 (or rabbit IgG control) was immunoprecipitated from M14-BMR cell lysate and blotted with the indicated antibodies. **(j)** Drug-treated (24h) M14-BMR cells (D/T=100nM/20nM, BV02=5μM) were lysed, 14-3-3 proteins (pan) were immunoprecipitated (mouse IgG served as a negative isotype control) and IPs blotted with the indicated antibodies. **(k)** M14-BMR cells expressing vector or IPTG-inducible *ABL1/2* shRNA were induced for 10 days with IPTG (1mM), cells lysed, 14-3-3 proteins (pan) immunoprecipitated (mouse IgG is an isotype control) and blotted with the indicated antibodies. **(l)** MAP3K1 (mouse IgG=isotype control) was immunoprecipitated from lysates described in (k) and blotted with antibodies.

Supplementary Figure 7

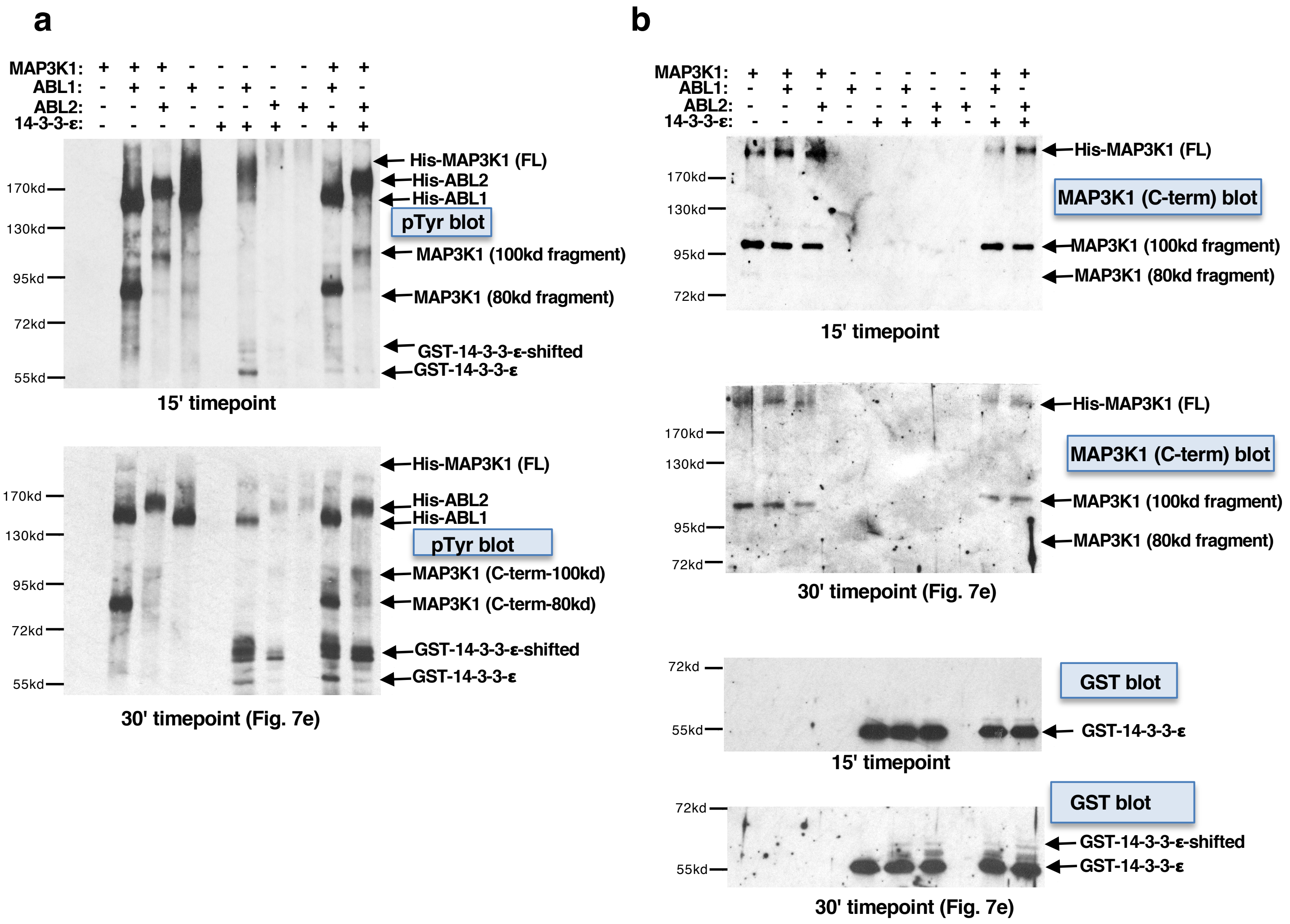

Supplementary Figure 7. ABL1/2 phosphorylate MAP3K1 and 14-3-3-ε. (a) Recombinant His-ABL1 or His-ABL2 (ThermoFisher) were incubated with recombinant full-length His-MAP3K1 (obtained with some breakdown products; Origene) and/or with GST-tagged 14-3-3-ε in kinase assay buffer for the indicated timepoints, and cold kinase reactions blotted with phospho-tyrosine (pTyr) antibody. A representative experiment from n=2 independent experiments is shown. **(b)** Blots from (a) were stripped and reprobed with antibody to MAP3K1 (top two blots; recognizes the C-terminus) or GST (light and dark film exposures are shown). A representative experiment from n=2 independent experiments is shown.

Supplementary Figure 8

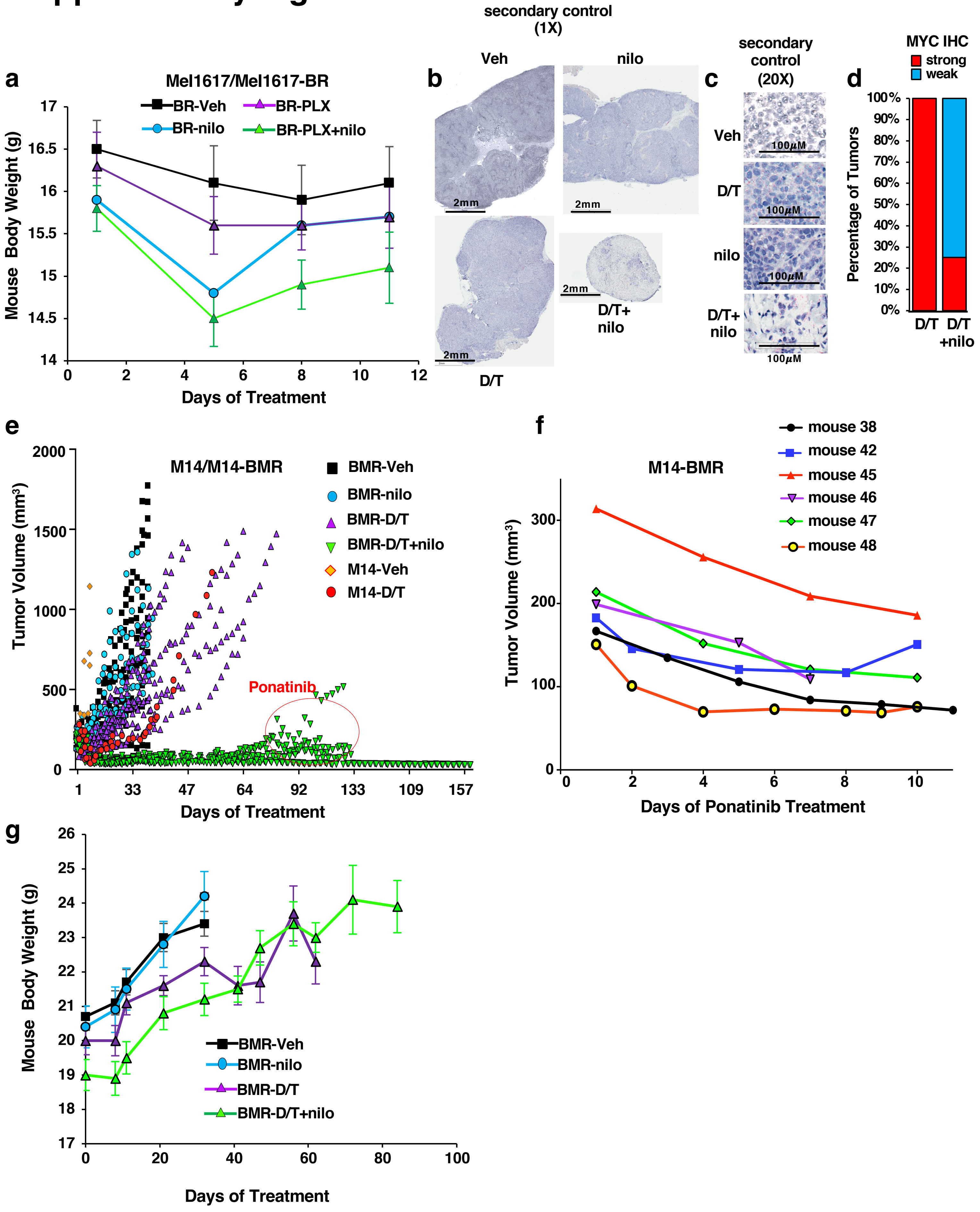

Supplementary Figure 8. Nilotinib reverses BRAFi and BRAFi/MEKi resistance, *in vivo*. (a) Body weights of animals harboring Mel1617-BR xenografts (see **Fig. 8a-c**); Mean$\pm$SEM. Vehicle (Veh, n=12), nilotinib (nilo, n=12), PLX4720 (PLX, n=12), or combination (n=11). No significant differences were noted between PLX and PLX+nilotinib treatment groups. **(b,c)** Secondary control staining for M14-BMR xenograft experiment (**see Fig. 8e**). **(b)** 1X magnification, **(c)** 20X magnification. **(d)** Graphical representation of scoring of the MYC staining (IHC) from **Fig. 8e.** Tumors removed on day 35 (n=4/group) were stained with MYC antibody, scored (intensity (1-3+) X percentage of positively staining tumor cells) blindly by a pathologist, categorized as weak ($\leq$1) or strong (>1), and percentages plotted. **(e)** M14-BMR xenograft experiment showing tumor growth of individual mice, following a cohort of triple-combination-treated mice (n=8) for 160d (see **Fig. 8d-g**). Red circle indicates triple-combination-treated mice who eventually relapsed and were subsequently treated with the 3rd generation ABL1/2 inhibitor, ponatinib (30mg/kg; n=6). A percentage of mice (2/8; 25%) never relapsed even after 160d of treatment. **(f)** Tumor growth curves for individual relapsed mice (e) treated with ponatinib for 10d. Ponatinib treatment had to be discontinued due to toxicity. **(g)** Mouse body weights for M14-BMR xenograft experiment (**Fig. 8d-g**). Mean$\pm$SEM is shown; no significant changes were noted. vehicle (Veh, n=12), nilotinib (nilo, n=12; 33mg/kg, BID), D/T (25mg/kg; 0.15mg/kg, n=12), or combination (n=11).

Supplementary Figure 9

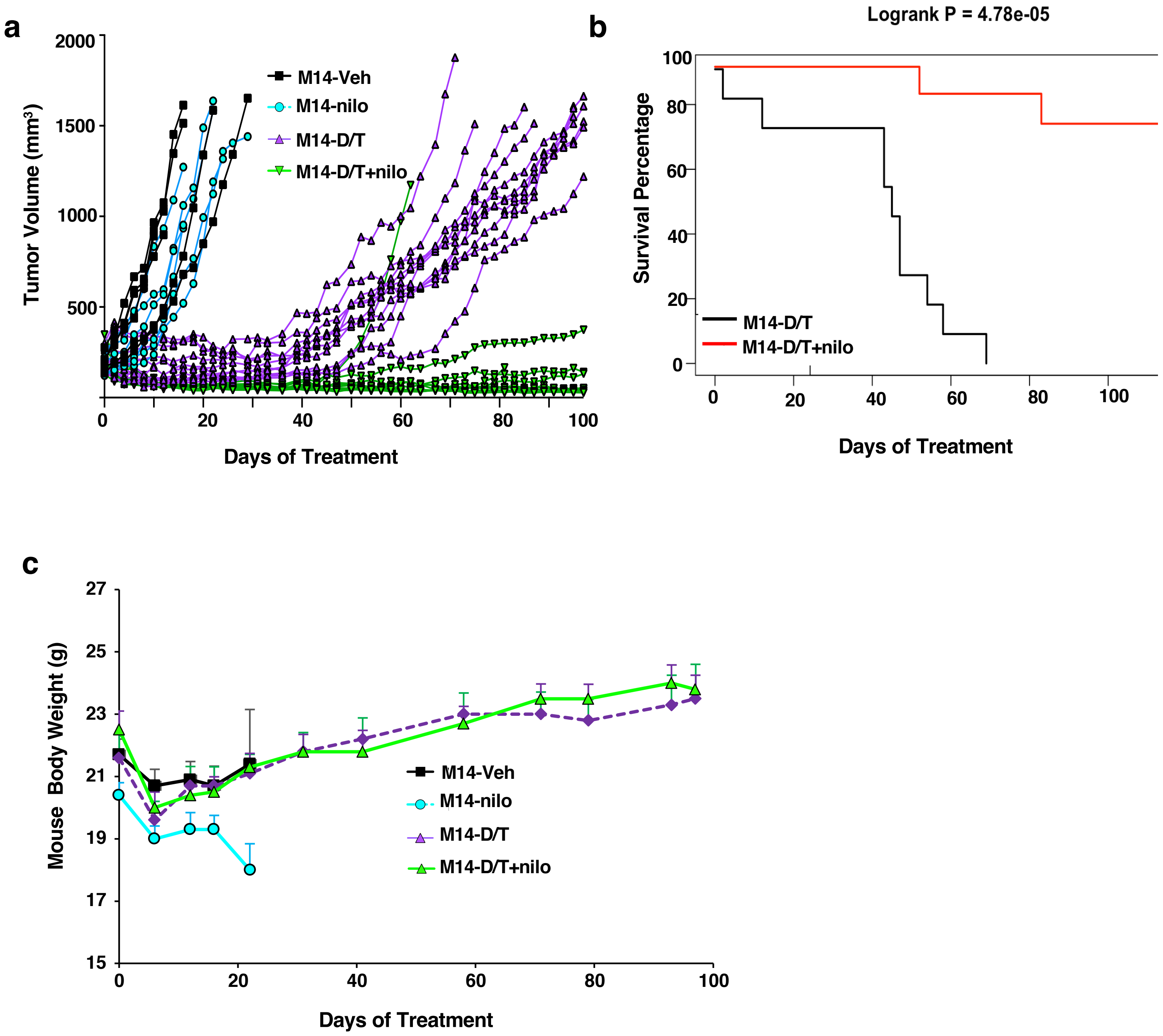

Supplementary Figure 9. Nilotinib prevents BRAFi/MEKi resistance from developing, *in vivo*. (a) Tumor growth curves for individual mice in the M14 prevention experiment (see **Fig. 8h,i**). Veh, n=5; nilo, n=6; D/T, n=11; DT+nilo, n=12. **(b)** Kaplan-Meier curve. Survival percentage endpoint is tumor size reaching 300mm^3. LogRank p=4.78e-05. **(c)** Mouse body weights for M14 prevention experiment (**Fig. 8h,i**); Mean±SEM is shown; no significant differences were noted. Veh, n=5; nilo, n=6; D/T, n=11; DT+nilo, n=12.

Supplementary Figure 10

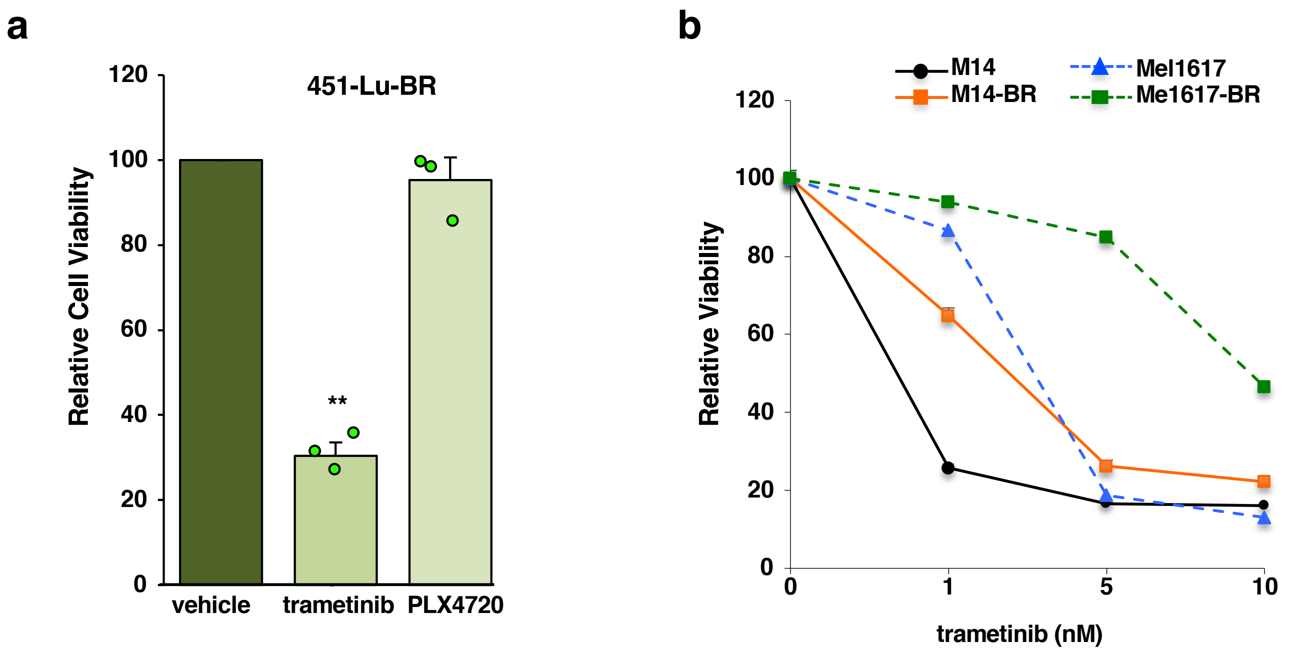

Supplementary Figure 10. 451-Lu-BR and M14-BR (but not Mel1617-BR) are sensitive to trametinib (MEKi). (a) 451-Lu-BR were treated with trametinib (2nM; MEKi) and/or PLX4720 (2.5μM) for 72h, and viability assessed with CellTiter Glo. Graph is Mean±SEM for n=3 biological replicates. p=0.0021 using a 2-sided, one sample t-test. **(b)** Parental or resistant Mel1617 or M14 cells were treated with the indicated doses of trametinib for 72h, and viability assessed with CellTiter Glo.

Supplementary Table 1

Treatment Group	LN Mets	
vehicle	2/12	
nilotinib	2/11	
dabrafenib+trametinib (D/T)	9/12	p=0.002
D/T+nilotinib	0/12	

Supplementary Table 1. Incidence of lymph node metastases in M14-BMR xenograft assay. The number of mice with palpable lymph node metastases in the resensitization experiment (Fig. 8d-g) are shown for each treatment group as compared to the number of animals in each group. Values were analyzed with Fisher's Exact Test (2-sided) using the Holm's procedure for multiple comparison adjustment. The p-value is for the D/T vs. D/T+nilotinib comparison.

Supplementary Table 2

Protein	clone/catalog #	Assay	Company	AB protocol	Tween %
14-3-3 (pan)	133233, -AC	IP; western	Santa Cruz	1:2000	0.20%
ABL1	clone 8E9, 56887	western	Santa Cruz	1:1000; 0.1%	0.10%
ABL1	K12-AC	kinase assay	Santa Cruz	1:400	N/A
ABL1-pT735	2864	western	Cell Signaling	1:1000, 5% BSA	0.10%
ABL2	5C6	western	Sigma	1:1000; 3% milk	0.05%
ABL2	clone 6D5	IP (coIPs)	Abnova	1:400	N/A
ABL2	rabbit serum	IP; kinase assay	lab specific	1:400	N/A
AKT	9272	western	Cell Signaling	1:5000; 5% BSA	0.10%
AKT (pS473)	4060	western	Cell Signaling	1:1000; 5% BSA	0.10%
beta actin	A5316, AC-74	western	MilliporeSigma	1:40,000	0.20%
beta tubulin	RB9249P	western	ThermoFisher	1:5000	0.10%
cleaved caspase-3	9664	western	Cell Signaling	1:5000, 5% milk	0.10%
cleaved PARP	5625	western	Cell Signaling	1:1000, 5% BSA	0.10%
CRAF	7267	western	Santa Cruz	1:500, 5% milk	0.10%
CRAF (pS338)	9427	western	Cell Signaling	1:1000; 5% BSA	0.10%
CRKL	3182	western	Cell Signaling	1:2000; 5% BSA	0.10%
CRKL (pY207)	3181	western	Cell Signaling	1:1000; 5% BSA	0.10%
ERK	610123	western	BD Biosciences	1:5000; 0.2% tween	0.20%
ERK (pT202/pY204)	RB9249P	western	Promega/VWR	1:10,000; 0.1% BSA	0.05%
FRA	AF4935	western	R&D	1:1000; 5% BSA	0.10%
FRA (pS265)	3880	western	Cell Signaling	1:1000; 5% BSA	0.10%
GAPDH	MA4300	western	ThermoFisher	1:50,000	0.20%
HA	12CA45	western	MilliporeSigma	1:5000, 5% milk	0.10%
JNK	AF1387	western	R&D	1:2000, 5% milk	0.10%
JNK (pY183/pY185)	AF1205	western	R&D	1:2000; 5% BSA	0.10%
lamin	clone 14, 05-714	western	MilliporeSigma	1:2000, 5% milk	0.10%
MAP3K1	AV48684	western	MilliporeSigma	1:1000, 2% milk	0.10%
MAP3K1	6850-AC	IP	Santa Cruz	1:400	N/A
MAP3K1	AP14770-PU-N	western	Origene	1:500, 5% milk	0.10%
MAP3K1 (pT1402)	SAB4504617	western	MilliporeSigma	1:1000; 5% BSA	0.10%
MAP3K8	MAB4586	western	R&D	1:1000, 3% milk	0.10%
MAP4K1	4472	western	Cell Signaling	1:1000, 5% BSA	0.10%
MEK	8727	western	Cell Signaling	1:5000; 5% BSA	0.10%
MEK (pS217/pS221)	9154	western	Cell Signaling	1:2000; 5% BSA	0.10%
MYC	clone 9E10, sc-40	western	Santa Cruz	1:1000; 5% BSA	0.10%
MYC	Y69, 790-4628	IHC	Roche/Ventana	24µg/ml	N/A
phospho-tyrosine	PY99	western	Santa Cruz	1:2000, 5% milk	0.20%
phospho-tyrosine	4G10	western	MilliporeSigma	1:10,000, 5% milk	0.20%
SRC	GD11, 05-184	western	Cell Signaling	1:2000; 5% milk	0.10%
SRC (pY416)	2101	western	Cell Signaling	1:1000, 5% BSA	0.10%
Secondary Antibodies					
anti-mouse HRP	PI31430	western	ThermoFisher	1:3000	0.10%
anti-rabbit HRP	NA934V	western	GE Healthcare	1:3000	0.10%
anti-goat HRP	805-035-180	western	Jackson Labs	1:3000	0.10%

Supplementary Table 2. Antibodies utilized and dilutions/procedures.

Supplementary Table 3

Sanger Sequencing	Primers
Forward Primer	5'-TGATGAGCAGCAGCGAAAGC-3'
Reverse Primer	5’-GAACACCACACCGCCATTGC-3’

Supplementary Table 3. Primers used for Sanger sequencing.
