# Supplementary material for: Combating acquired resistance to MAPK inhibitors in melanoma by targeting Abl1/2-mediated reactivation of MEK/ERK/MYC signaling
Source: Nat Commun. 2020 Oct 29;11:5463. doi: 10.1038/s41467-020-19075-3 (PMC7596241; doi:10.1038/s41467-020-19075-3)
Supplement: Supplementary file 3 — Description of Additional Supplementary Files [file 41467_2020_19075_MOESM3_ESM.pdf]

**Description of Additional Supplementary Files**

**Supplementary Data 1:** Whole Exome Sequencing for M14, M14-BR, M14-BMR, Mel1617, and Mel1617-BR comparing resistant lines to parental lines (subtracting out mutations that were present in parental lines). Mutations observed in resistant lines are shown.

**Supplementary Data 2:** Whole Exome Sequencing for 451-Lu and 45-Lu-BR-resistant samples, comparing resistant line to parental line (subtracting out mutations that were present in the parental line). Mutations observed in resistant lines are shown.

**Supplementary Data 3-8:** Whole Exome Sequencing for Mel1617-BR responding and non-responding xenografts samples (two tumors apiece).

**Supplementary Data 3:** Unique gene mutations identified in both responding tumors.

**Supplementary Data 4:** Unique gene mutations identified in both non-responding tumors.

**Supplementary Data 5:** Gene mutations in common between all tumors.

**Supplementary Data 6:** Gene mutations in non-responding tumor-mouse #38.

**Supplementary Data 7:** gene mutations in non-responding tumor mouse #48.

**Supplementary Data 8:** gene mutations in responding tumor-mouse #45.

**Supplementary Data 9:** gene mutations in responding tumor mouse #46
