# Supplementary material for: Combating acquired resistance to MAPK inhibitors in melanoma by targeting Abl1/2-mediated reactivation of MEK/ERK/MYC signaling
Source: Nat Commun. 2020 Oct 29;11:5463. doi: 10.1038/s41467-020-19075-3 (PMC7596241; doi:10.1038/s41467-020-19075-3)
Supplement: Supplementary file 13 — Reporting summary [file 41467_2020_19075_MOESM13_ESM.pdf]

nature research

Corresponding author(s): Rina Plattner

Last updated by author(s): Sep 10, 2020

Reporting Summary

Nature Research wishes to improve the reproducibility of the work that we publish. This form provides structure for consistency and transparency in reporting. For further information on Nature Research policies, see Authors & Referees and the Editorial Policy Checklist .

Statistics

For all statistical analyses, confirm that the following items are present in the figure legend, table legend, main text, or Methods section.

n/a	Confirmed	
☐	☒	The exact sample size (*n*) for each experimental group/condition, given as a discrete number and unit of measurement
☐	☒	A statement on whether measurements were taken from distinct samples or whether the same sample was measured repeatedly
☐	☒	The statistical test(s) used AND whether they are one- or two-sided<br>*Only common tests should be described solely by name; describe more complex techniques in the Methods section.*
☐	☒	A description of all covariates tested
☐	☒	A description of any assumptions or corrections, such as tests of normality and adjustment for multiple comparisons
☐	☒	A full description of the statistical parameters including central tendency (e.g. means) or other basic estimates (e.g. regression coefficient) AND variation (e.g. standard deviation) or associated estimates of uncertainty (e.g. confidence intervals)
☐	☒	For null hypothesis testing, the test statistic (e.g. *F*, *t*, *r*) with confidence intervals, effect sizes, degrees of freedom and *P* value noted<br>*Give P values as exact values whenever suitable.*
☒	☐	For Bayesian analysis, information on the choice of priors and Markov chain Monte Carlo settings
☒	☐	For hierarchical and complex designs, identification of the appropriate level for tests and full reporting of outcomes
☒	☐	Estimates of effect sizes (e.g. Cohen's *d*, Pearson's *r*), indicating how they were calculated

Our web collection on statistics for biologists contains articles on many of the points above.

Software and code

Policy information about availability of computer code

Data collection	Sequencing reads were trimmed and filtered using Trimmomatic (V0.39), then aligned to human reference genome b37/hg19 using BWA (V. 0.7.17). PCR duplicates were removed using Picard (v2.20.2; http://broadinstitute.github.io/picard/).
Data analysis	The Genome Analysis Toolkit (GATK v4.1.2.0) was used for base quality score recalibration. For comparisons between resistant lines and their parental counterparts, somatic mutations present in the resistant line but not in the parental line were detected using Mutect2 (part of the GATK toolkit) tumor/normal mode with the parental line as the reference. Mutations passing the contamination filter and orientation bias filter were annotated (Oncotator, v1.9.9.0). RNAseq/microarray datasets for Fig. 1d/Supplementary Fig. 1f were downloaded from the GEO database (accession #: 50535, 50509, 77940), and bam files for EGSA1000992 were kindly provided by Drs. Lawrence Kwong (MD Anderson, Houston, TX). A two-sided binomial was utilized to analyze whether ABL1 mRNA was elevated or downregulated following resistance. In Fig. 6b, three mRNASeq datasets were utilized: GSE50535, GSE77940 and EGAS00001000992. Since the sample sizes of these datasets were limited, in order to increase sample size and therefore statistical power, we combined datasets with the R package "sva", which removes batch effects using an empirical Bayesian framework. After combining the datasets, Spearman's rank-order correlation analysis was performed to test the correlations between ABL1 and MYC before and after treatment, respectively. For Supplementary Fig. 5c, RNASeq data were downloaded from Genomic Data Commons (GDC) for TCGA Skin Cutaneous Melanoma data (access date, November 2019) and normalized to Transcripts Per Kilobase Million (TPM). Spearman's correlation coefficients were used to quantify the correlation between ABL1 and MYC mRNAs. ImageJ (V1.44o) was used to quantitate blots. 4 Peaks free software for Mac (2004-2015) was used to view Sanger sequencing .ab files.

For manuscripts utilizing custom algorithms or software that are central to the research but not yet described in published literature, software must be made available to editors/reviewers. We strongly encourage code deposition in a community repository (e.g. GitHub). See the Nature Research guidelines for submitting code & software for further information.

Data

Policy information about availability of data

All manuscripts must include a data availability statement. This statement should provide the following information, where applicable:

- Accession codes, unique identifiers, or web links for publicly available datasets
- A list of figures that have associated raw data
- A description of any restrictions on data availability

NextGeneration sequencing (WES) data have been deposited in the NCBI/SRA database under the accession code (PRJNA639552).

STUDY: PRJNA639552
SAMPLE: M14 parental (SAMN15239022)
EXPERIMENT: M14-WES (SRX8550107)
RUN: M14-P_S5_L000_R1_001.fastq.gz (SRR12017626)
SAMPLE: M14-BR (SAMN15239023)
EXPERIMENT: M14-BR-WES (SRX8550108)
RUN: M14-BR_S6_L000_R1_001.fastq.gz (SRR12017625)
SAMPLE: M14-BMR (SAMN15239024)
EXPERIMENT: M14-BMR-WES (SRX8550109)
RUN: M14-BMR_S7_L000_R1_001.fastq.gz (SRR12017624)
SAMPLE: Mel1617 parental (SAMN15239025)
EXPERIMENT: Mel-WES (SRX8550110)
RUN: Mel1617-P_S2_L000_R1_001.fastq.gz (SRR12017623)
SAMPLE: Mel1617-BR (SAMN15239026)
EXPERIMENT: MelBR-WES (SRX8550111)
RUN: Mel1617-BR_S3_L000_R1_001.fastq.gz (SRR12017622)
SAMPLE: 451-Lu parental (SAMN15239028)
EXPERIMENT: 451-WES (SRX8550113)
RUN: 451-Lu-P_S1_L000_R1_001.fastq.gz (SRR12017620)
SAMPLE: 451-Lu-BR (SAMN15239029)
EXPERIMENT: 451BR-WES (SRX8550114)
RUN: 451-Lu-BR_S1_L000_R1_001.fastq.gz (SRR12017619)

https://nam04.safelinks.protection.outlook.com/?url=https%3A%2F%2Fwww.ncbi.nlm.nih.gov%2FTraces%2Fstudy%2F%3Facc%3DPRJNA639552&data=02%7C01%7CRina.Plattner%40uky.edu%7Cefbd7ff441334045f3df08d85166bb99%7C2b30530b69b64457b818481cb53d42ae%7C0%7C0%7C637348848961100634&sdata=vHZhMJXq%2Fe3EqqMdZmQxxZD4IkbAfGo1WzNysiu5wWc%3D&reserved=0

The source data underlying all Figures (1-8) and Supplementary Figures (1-10) are provided as a Source Data file. All the other data supporting the findings of this study are available within the article and its supplementary information files and from the corresponding author upon reasonable request.

Databases used in the study are GEO: https://www.ncbi.nlm.nih.gov/geo/query/acc.cgi?acc=GSE50535, https://www.ncbi.nlm.nih.gov/geo/query/acc.cgi?acc=GSE50509, https://www.ncbi.nlm.nih.gov/geo/query/acc.cgi?acc=GSE77940
https://www.ncbi.nlm.nih.gov/geo/query/acc.cgi?acc=GSE65185

Genomic Data Commons-GDC (TCGA data): https://portal.gdc.cancer.gov/projects/TCGA-SKCM

European Genome Phenome Archive (data kindly provided by Dr. Lawrence Kwong, MD Anderson): https://www.ebi.ac.uk/ega/datasets

cBioportal was used to determine whether particular mutations were present in patient samples.
PhosphoSite was used to query the presence of particular phosphorylation sites in proteins.

Field-specific reporting

Please select the one below that is the best fit for your research. If you are not sure, read the appropriate sections before making your selection.

☒ Life sciences ☐ Behavioural & social sciences ☐ Ecological, evolutionary & environmental sciences

For a reference copy of the document with all sections, see nature.com/documents/nr-reporting-summary-flat.pdf

Life sciences study design

All studies must disclose on these points even when the disclosure is negative.

Sample size	For animal studies, sample size was calculated based on pilot experiments or previous data. Assuming a 50% or 75% reduction in tumor volume for single agent and combination groups, respectively, compared to a vehicle mean=600mm3 (SD=225mm3), twelve mice/group provides 85% power and 5% alpha to detect the difference between combination and single agents based on ANOVA adjusted for multiple comparisons. Sample size calculations were not performed for in vitro experiments. Three independent experiments were performed for most figures. If significance was not reached, or we wanted corroboration of the results by an independent lab member, an additional biological experiment was performed and data expressed as n=4 or n=5.
Data exclusions	Animals who died of experiment-independent causes (e.g. gavage injury) at early points in the experiment were removed from the study, resulting in some groups containing 11 rather than 12 animals (or 5 vs. 6 for controls).
Replication	Most in vitro experiments were performed at least 3 independent times. All values are shown within the graphs. Cell Titer Glo assays involved >3 biological replicates performed in triplicate (3 internal replicates). For in vivo experiments, individual tumor measurements are shown in graphs in Supplementary information.
Randomization	For animal experiments, mice containing tumors (mean volume=200mm3) were randomized to one of four groups (e.g. vehicle, nilotinib, D/T, D/T+nilotinib). For in vitro studies, all cells from multiple dishes were combined and then plated into wells that were treated with various drugs. Thus, all treatment groups came from the same cell stock. Biological independent experiments were performed on independent aliquots of cells thawed from the liquid nitrogen freezer.
Blinding	Laboratory personnel were blind to animal randomization to drug treatment, which was performed by the PI. Laboratory personnel could not be blinded during the experiment as they needed to know which groups to treat with which drugs. Data analyses was performed by the Biostatistics and Bioinformatics Shared Resource facility who were blinded to experimental groups. All IHC was blindly scored by pathologist, Dr. Dana Richards.

Reporting for specific materials, systems and methods

We require information from authors about some types of materials, experimental systems and methods used in many studies. Here, indicate whether each material, system or method listed is relevant to your study. If you are not sure if a list item applies to your research, read the appropriate section before selecting a response.

Materials & experimental systems

n/a	Involved in the study	
☐	☒	Antibodies
☐	☒	Eukaryotic cell lines
☒	☐	Palaeontology
☐	☒	Animals and other organisms
☒	☐	Human research participants
☒	☐	Clinical data

Methods

n/a	Involved in the study	
☒	☐	ChIP-seq
☒	☐	Flow cytometry
☒	☐	MRI-based neuroimaging

Antibodies

Antibodies used

Protein, clone/catalog #, AB dilution
14-3-3 (pan), 133233, -AC, 1:2000
ABL1, clone 8E9, 1:1000
ABL1, K12-AC, kinase assay-1:400 for IP
ABL1-pT735, 2864, 1:1000
ABL2 6D5, 1:400, IP
ABL2, 5C6, 1:1000
ABL2, rabbit serum, kinase assay, 1:160
AKT, 9272, 1:5000
AKT (pS473), 4060, 1:1000
beta actin, AC-74, A5316, 1:40,000
beta tubulin, RB9249P, 1:5000
cleaved caspase-3, 9664, 1:5000
cleaved PARP, 5625, 1:1000
CRAF, 7267, 1:500
CRAF (pS338), 9427, 1:1000
CRKL, 3182, 1:2000
CRKL (pY207), 3181, 1:1000

ERK, 610123, 1:5000
ERK (pT202/pY204), RB9249P, 1:10,000
FRA, AF4935, 1:1000
FRA (pS265),3880, 1:1000
GAPDH, MA4300, 1:50,000
HA, 12CA5 , 1:5000
JNK, AF1387, 1:2000
JNK (pY183/pY185), AF1205, 1:2000
lamin, clone 14, 1:2000
MAP3K1, AV48684, 1:1000
MAP3K1, 17820-AC,
MAP3K1, AP14770-PU-N, 1:500
MAP3K1 (pT1402), SAB4504617, 1:1000
MAP3K8, MAB4586, 1:1000
MAP4K1, 4472, 1:1000
MEK, 8727, 1:5000;
MEK (pS217/pS221), 9154, 1:2000
MYC, clone 9E10, sc-40, 1:1000
MYC, 32072, 1:100, IHC
phospho-tyrosine, PY99, 1:2000
phospho-tyrosine, 4G10, 1:10,000
SRC, GD11, 1:2000; SRC (pY416), 2101, 1:1000

Secondary Antibodies
anti-mouse HRP, PI31430, 1:3000
anti-rabbit HRP, NA934V, 1:3000
anit-goat HRP 805-035-180, 1:3000

Validation

Primary Antibodies.
Below are validation statements from manufacturers as well as validation performed in-house and by other investigators.
14-3-3 (pan), 133233, -AC, IP; western, Santa Cruz; https://www.scbt.com/p/pan-14-3-3-antibody-b-8?requestFrom=search
ABL1, clone 8E9, western, Santa Cruz; https://www.scbt.com/p/c-abl-antibody-8e9?requestFrom=search; knockdown experiments in this manuscript.
ABL1, K12-AC, kinase assay, Santa Cruz; validated by Plattner et al. Genes Dev 13:2400-2411, 1999. https://www.scbt.com/p/c-abl-antibody-k-12
ABL1-pT735, 2864, western, Cell Signaling; https://www.cellsignal.com/products/primary-antibodies/phospho-c-abl-thr735-antibody/2864
ABL2, 5C6, western, Sigma. Validated by shRNA; Jain et al. 36(32):4585-4596, 2017; knockdown experiments in this manuscript
ABL2, clone 6D5, IP, western (coIPs), Abnova; Validated by Hoj et al. Cell Reports 29:3421-3434, 2019.
ABL2, rabbit serum, IP; kinase assay, lab specific; validated by knockdown and described in Finn et al. Nature Neuroscience 6 (7):717-723, 2003.
AKT, 9272, western, Cell Signaling; https://www.cellsignal.com/products/primary-antibodies/akt-antibody/9272
AKT (pS473), 4060, western, Cell Signaling; https://www.cellsignal.com/products/primary-antibodies/phospho-akt-ser473-d9e-xp-rabbit-mab/4060
beta actin, AC-74, A5316, western, MilliporeSigma; https://www.sigmaaldrich.com/catalog/product/sigma/a5316?lang=en&region=US&gclid=Cj0KCQjw7sz6BRDYARIsAPHzrNJqKYO1uNwP_AhT4WzuW5iO79vMVZVf65l8aWMt_YPbbFl0oIBy7VAaAiSIEALw_wcB
beta tubulin, RB9249P, western, ThermoFisher; https://assets.thermofisher.com/TFS-Assets/APD/Specification-Sheets/D12587~.pdf
cleaved caspase-3, 9664, western, Cell Signaling; https://www.cellsignal.com/products/primary-antibodies/cleaved-caspase-3-asp175-5a1e-rabbit-mab/9664
cleaved PARP, 5625, western, Cell Signaling, https://www.cellsignal.com/products/primary-antibodies/cleaved-parp-asp214-d64e10-xp-rabbit-mab/5625
CRAF, 7267, western, Santa Cruz; https://www.scbt.com/p/raf-1-antibody-e-10?requestFrom=search
CRAF (pS338), 9427, western, Cell Signaling; https://www.cellsignal.com/products/primary-antibodies/phospho-c-raf-ser338-56a6-rabbit-mab/9427
CRKL, 3182, western, Cell Signaling; https://www.cellsignal.com/products/primary-antibodies/crkl-32h4-mouse-mab/3182
CRKL (pY207), 3181, western, Cell Signaling; https://www.cellsignal.com/products/primary-antibodies/phospho-crkl-tyr207-antibody/3181
ERK, 610123, western, BD Biosciences
ERK (pT202/pY204), RB9249P, western, Promega/VWR; https://us.vwr.com/store/product?keyword=PAV8031
FRA, AF4935, western, R&D; https://www.rndsystems.com/products/human-fra-1-antibody_af4935
FRA (pS265), 3880, western, Cell Signaling; https://www.cellsignal.com/products/primary-antibodies/phospho-fra1-ser265-antibody/3880
GAPDH, MA4300, western, ThermoFisher; https://www.thermofisher.com/antibody/product/GAPDH-Antibody-clone-6C5-Monoclonal/AM4300

HA, 12CA5 , western, MilliporeSigma; https://www.sigmaaldrich.com/catalog/product/roche/roaha?lang=en&region=US&gclid=Cj0KCQjw7sz6BRDYARIsAPHzrNLCVqmEoT43lr3mFomMXA03h6bXMcKDW0DHuuP8tWhRTmdoyHGC4a8aArpaEALw_wcB
JNK, AF1387, western, R&D; https://www.rndsystems.com/products/human-mouse-rat-jnk-pan-specific-antibody_af1387
JNK (pY183/pY185), AF1205, western, R&D; https://www.rndsystems.com/products/human-mouse-rat-phospho-jnk-t183-y185-antibody_af1205
lamin, clone 14, 05-714, western, MilliporeSigma; https://www.sigmaaldrich.com/catalog/product/mm/05714?lang=en&region=US
MAP3K1, AV48684, western, MilliporeSigma; https://www.sigmaaldrich.com/catalog/product/sigma/av48684?lang=en&region=US; also validated by siRNA in this manuscript.
MAP3K1, 17820-AC, IP, Santa Cruz, https://www.scbt.com/p/mek-kinase-1-antibody-f-11?requestFrom=search
MAP3K1, AP14770-PU-N, western, Origene; https://www.origene.com/catalog/antibodies/primary-antibodies/ap14770pu-n/mekk1-map3k1-rabbit-polyclonal-antibody.
MAP3K1 (pT1402), SAB4504617, western, MilliporeSigma; https://www.sigmaaldrich.com/catalog/product/sigma/sab4501862?lang=en&region=US
MAP3K8, MAB4586, western, R&D; https://www.rndsystems.com/search?keywords=map3k8
MAP4K1, 4472, western, Cell Signaling; https://www.cellsignal.com/products/primary-antibodies/hpk1-antibody/4472; also validated by siRNA in this manuscript.
MEK, 8727, western, Cell Signaling; https://www.cellsignal.com/products/primary-antibodies/mek1-2-d1a5-rabbit-mab/8727
MEK (pS217/pS221), 9154, western, Cell Signaling; https://www.cellsignal.com/products/primary-antibodies/mek1-2-d1a5-rabbit-mab/8727
MYC, clone 9E10, 40, western, Santa Cruz; https://www.scbt.com/p/c-myc-antibody-9e10?requestFrom=search
MYC, Y69, #790-4628; IHC, Ventana/Roche; prediluted 24ug/ml; https://pim-eservices.roche.com/eLD/api/downloads/f90f3091-1d36-ea11-fc90-005056a71a5d?countryIsoCode=us
phospho-tyrosine, PY99, 7020, western, Santa Cruz; https://www.scbt.com/p/p-tyr-antibody-py99?requestFrom=search
phospho-tyrosine, 4G10, western, EMD MilliporeSigma; https://www.emdmillipore.com/US/en/product/Anti-Phosphotyrosine-Antibody-clone-4G10,MM_NF-05-321X
SRC, GD11, 05-184, western, MilliporeSigma; https://www.emdmillipore.com/US/en/product/Anti-Src-Antibody-clone-GD11,MM_NF-05-184
SRC (pY416), 2101, western, Cell Signaling; https://www.cellsignal.com/products/primary-antibodies/phospho-src-family-tyr416-antibody/2101

Secondary Antibodies.
anti-mouse HRP, PI31430, western, ThermoFisher; https://www.thermofisher.com/antibody/product/Goat-anti-Mouse-IgG-H-L-Secondary-Antibody-Polyclonal/31430
anti-rabbit HRP, NA934V, western, GE Healthcare; https://us.vwr.com/store/product/16776610/anti-igg-donkey-polyclonalantibody-hrp-horseradish-peroxidase
anti-goat HRP, 805-035-180, western, https://www.jacksonimmuno.com/catalog/products/805-035-180

Eukaryotic cell lines

Policy information about cell lines

Cell line source(s)

M14 cells were obtained from NCI (NCI-60 panel). Mel1617, Mel1617-BR, 451-Lu, 451-Lu-BR, and WM164 cells were obtained from Meenhard Herlyn (Wistar Institute, Philadelphia, PA) via an MTA. Mel1617-BR and 451-Lu-BR were described as clonal lines that were established by culturing cells in increasing doses of the BRAFi, SB590885, followed by isolating individual clones. STR profiling analysis was repeated on the four cell lines in 2018 (Wistar Institute). M14-BR, M14-BMR, and Mel1617-BMR were generated in our lab from M14 and Mel1617 parental cells by culturing cells in increasing concentrations of PLX4720 (M14-BR; up to 1uM) or dabrafenib+trametinib (M14-BMR; Mel1617-BMR up to 100nM/20nM) until resistant at the indicated dosage (about 6 months), and pooling resistant clones. 293T cells, used for heterologous gene expression, were obtained from ATCC.

Authentication

Mel1617, 451, and WM164 lines were obtained from M. Herlyn and all 3 cell lines were authenticated by the Wistar Institute using short tandem repeat (STR) profiling. M14 cells were obtained from NCI (NCI-60 panel) and were used to make resistant lines within 5 passages of thawing; thus, their identity was not further authenticated. Resistant counterparts (-BR, -BMR) were made from the parental lines by exposure to increasing concentration of drugs. 293T cells, obtained from ATCC and used solely for heterologous protein expression, were not authenticated.

Mycoplasma contamination

All cell lines are periodically tested using MycoAlert (Lonza) and all cell lines tested negative.

Commonly misidentified lines
(See ICLAC register)

M14. This line has been mis-identified as a breast cancer line (MDA-MB-435); however, STR profiling has confirmed its melanoma origin. "STR profiling showed that this cell line has a shared donor origin with MDA-MB-435 and M14. The identity of these two cell lines was investigated further by ICLAC; all derivatives correspond to the M14 melanoma cell line and its donor (Korch et al, 2018)." This cell line was used because it is extremely sensitive to BRAFi and has high levels of ABL1 and ABL2 kinase activities and thus, is a good model for testing our hypotheses. All data obtained with this cell line were also

confirmed in additional cell lines.

Animals and other organisms

Policy information about studies involving animals; ARRIVE guidelines recommended for reporting animal research

Laboratory animals	Nu/Nu mice (female, 6 weeks old) were used for the M14-BMR study, while SCID/beige mice (female, 6 weeks old) were used for the Mel1617-BR study.
Wild animals	No wild animals were used for this study.
Field-collected samples	No field collected samples were used for this study.
Ethics oversight	All animal experiments were approved by the University of Kentucky Institutional Care and Use Committee (Protocol #00946M2005), and experiments were performed in accordance within University and NIH guidelines.

Note that full information on the approval of the study protocol must also be provided in the manuscript.
